# Reducing Disease Activity of Inflammatory Bowel Disease by Consumption of Plant-Based Foods and Nutrients

**DOI:** 10.3389/fnut.2021.733433

**Published:** 2021-12-09

**Authors:** Christian S. Antoniussen, Henrik H. Rasmussen, Mette Holst, Charlotte Lauridsen

**Affiliations:** ^1^Department of Clinical Medicine, Faculty of Medicine, Aalborg University, Aalborg, Denmark; ^2^Department of Gastroenterology, Center for Nutrition and Bowel Disease, Aalborg University Hospital, Aalborg, Denmark; ^3^Department of Animal Science, Faculty of Technical Sciences, Aarhus University, Foulum, Denmark

**Keywords:** immunity, microbiota, disease activity, plant-based foods, inflammatory bowel disease

## Abstract

Inflammatory bowel disease is a chronic and recurring inflammatory condition of the gastrointestinal tract encompassing ulcerative colitis and Crohn's disease. Although the pathogenesis of inflammatory bowel disease remains to be fully elucidated, environmental factors such as diet are believed to play a pivotal role in the onset and management of inflammatory bowel disease. Diet is thought to play an essential role in intestinal inflammation due to its regulatory effects on the microbiota, gut immune system, and epithelial barrier function. Although the evidence remains insufficient to draw firm conclusions on the role of specific dietary components in gastrointestinal diseases, studies have suggested that a Western diet with high intakes of total fats, omega-6 fatty acids, and meat have been associated with intestinal inflammation and relapse of inflammatory bowel disease. In contrast to a Western diet, plant-based diets often result in a reduced intake of total fats and meats and an increased intake of plant fibers which may contribute to reduced intestinal inflammation. This review critically examines the influence of plant-based dietary components on the clinical disease course of inflammatory bowel disease. Furthermore, this review discusses the benefits and possible limitations of plant-derived dietary components in the treatment of inflammatory bowel disease while addressing the principal type of disease and the anatomic site of inflammation within the gastrointestinal tract. Finally, this review points out important directions for future research on the role of diet in inflammatory bowel disease. A better understanding of the role of diet and intestinal inflammation may pave the way for novel dietary interventions and specific foods- or food supplements, which can support the treatment of inflammatory bowel disease.

## Introduction

**Figure 1:**
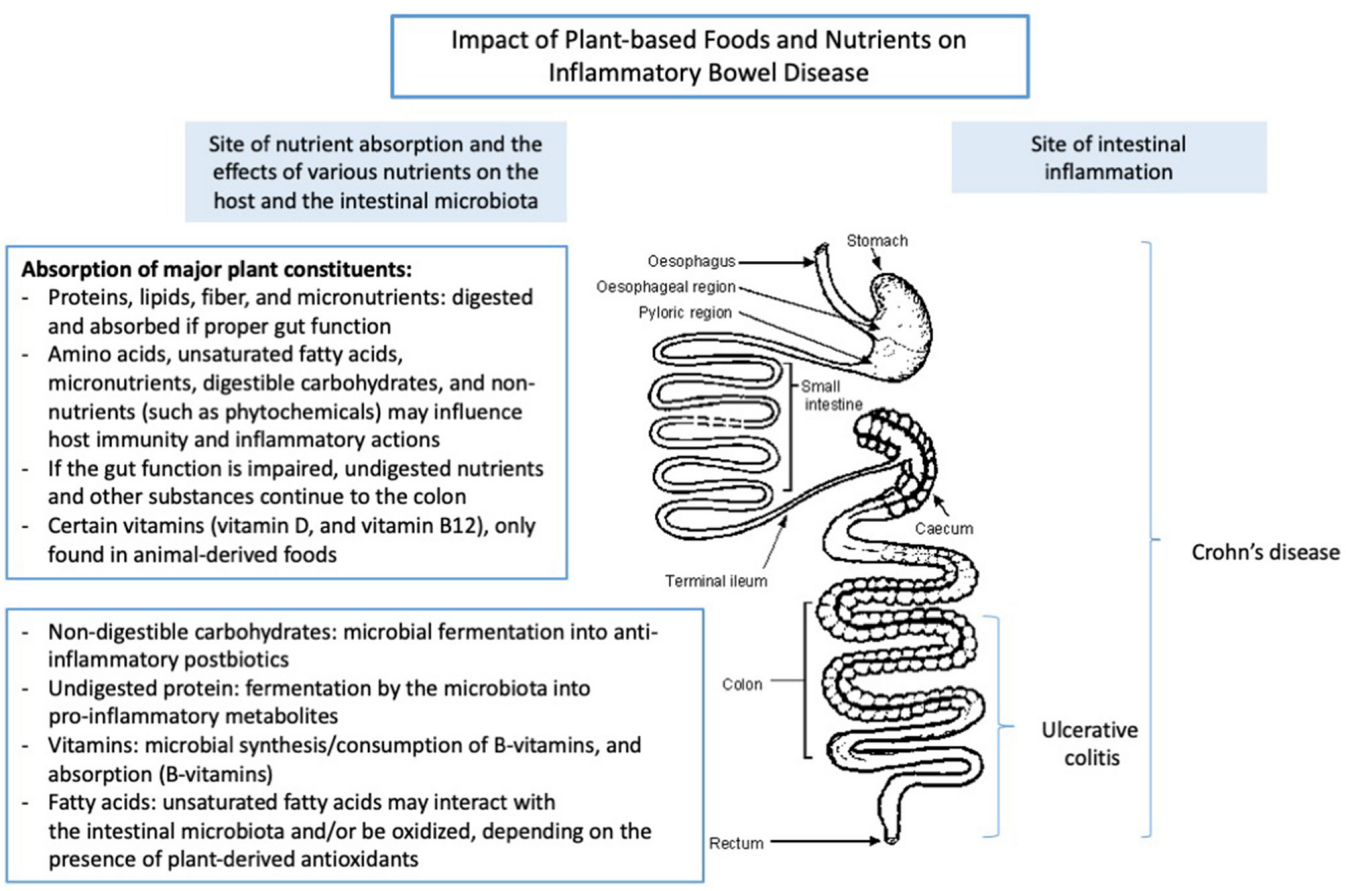
The impact of plant-based foods and nutrients on the intestine. Adapted from Lauridsen et al. (15).

Crohn's disease (CD) and ulcerative colitis (UC) are chronic relapsing inflammatory bowel diseases (IBD) (1, 2). Although CD and UC are characterized by intestinal inflammation, they have distinct pathological characteristics. CD is characterized by segmental transmural inflammation of the intestinal mucosa that may occur anywhere in the gastrointestinal tract, most frequently affecting the ileum, while UC is described as a non-transmural inflammatory disorder of the colon extending from the rectum to the proximal colon (3). The medical treatment of IBD is initiated to reduce its disease activity and to induce and maintain clinical remission (3). Although the etiology of IBD is not fully understood, diet has been suggested to play a pivotal role in its pathogenesis and course of the disease. Various dietary components may impact on the disease course due to their regulatory effects on the intestinal microbiota, mucosal barrier function, nutritional status, and intestinal immunity (4–7). In particular, so-called Western diets and animal-based diets, high in animal-derived protein, saturated fats, altered omega-6 to omega-3 ratio, and diets low in fruits and vegetables may contribute to intestinal inflammation. These diets may contribute to intestinal inflammation through alterations in the intestinal microbiota, decreased production of immunoregulatory metabolites such as short-chain fatty acids (SCFAs) including increased production of microbial proteolytic fermentation metabolites, which may comprise the colonic epithelial cell structure and cause damage to the intestinal barrier function (8–10). Although the nutrient content and composition of plant foods vary greatly, the consumption of plant-based diets often result in a reduced intake of saturated fatty acids (SFAs) and animal-derived dietary protein and a concomitant higher intake of non-digestible carbohydrates (NDCs) and phytochemicals due to intakes of fruits and vegetables. Together, this may reduce intestinal inflammation and disease activity through enhanced production of immunoregulatory postbiotics, down-regulation of pro-inflammatory cytokines, and increased microbial diversity (11–13). Therefore, dietary changes from an animal-based diet toward a diet primarily based on plant foods may possess the ability to inhibit intestinal inflammation in active IBD, reduce its disease severity and contribute to the maintenance of clinical remission. However, despite the potential beneficial effects of plant-based diets in IBD, these diets may provide insufficient amounts of high-quality protein and some micronutrients, which may be essential to maintain a proper nutritional status. Similarly, plant-based diets may also provide inadequate amounts of long-chain polyunsaturated fatty acids such as Docosahexaenoic acid (DHA) and Eicosapentaenoic acid (EPA) found in high concentrations in marine food sources that may regulate various physiological aspects of intestinal inflammation (14). With this knowledge, we aim to examine the benefits and possible limitations of consuming various plant-based dietary components in relation to the central question: “Is it possible to induce or maintain clinical remission of IBD by eating a plant-based diet”? Similarly, we aim to discuss the potential role of plant-based nutrients and dietary components in the treatment of IBD, while the anatomic site of inflammation within the gastrointestinal tract is taken into consideration (Figure 1).

## Plant-Derived Dietary Protein

### Importance of Sufficient Protein Consumption in Patients With Inflammatory Bowel Disease

Adequate dietary protein consumption plays an essential role in IBD patients because of the increased risk of disease-related malnutrition found in these patients (16, 17). Depending on the anatomic site of inflammation within the gastrointestinal tract, patients with IBD often experience nutrient malabsorption, nutrient depletion, including periods of anorexia, which contribute to an increased risk of malnutrition and loss of lean mass. This may occur due to the increased circulation of inflammatory cytokines, intestinal loss of nutrients, increased rates of protein turnover, proteolytic catabolic responses, and increased requirements of protein and amino acids (AAs) during active inflammation (17, 18). Due to the increased requirements of protein during active disease, the European Society of Clinical Nutrition and Metabolism (ESPEN) recommends increased consumption of dietary protein corresponding to 1.2–1.5 g/kg/day in adult IBD patients with active disease and approximately 1.0 g/kg/day in adult IBD patients in clinical remission (18). These recommendations are formulated to prevent malnutrition, including loss of muscle mass often observed in IBD patients (18). Despite these recommendations, a recent study of the habitual dietary intake of IBD patients demonstrated that many patients with IBD, irrespective of disease phenotype and disease activity, had a insufficient protein intake (19). Based on these observations, more effort must be made to ensure an adequate protein intake to prevent malnutrition in IBD patients. Beyond the overall amount of protein, dietary proteins including several AAs are suggested to play an essential role in maintaining intestinal homeostasis (20). Based on experimental models and animal studies of IBD, dietary protein, including AAs such as L-glutamine, L-arginine, and essential amino acids (EAAs) like tryptophan and lysine, have been suggested to impact the composition and function of the microbiota (20). In addition, these AAs have shown the ability to strengthen the intestinal mucosal barrier function, modulate the intestinal immune function, enhance mucosal healing and potentially attenuate intestinal inflammation and hence, impact disease activity in IBD (21). Together, these observations highlight the importance of ensuring a sufficient intake of dietary protein in IBD patients. Similarly, there seems to be a potential for AA supplementation in IBD to attenuate intestinal inflammation. However, the efficacy of dietary supplementation with AAs on inflammation and disease activity in humans remains to be determined (22, 23).

### Nutritional Quality of Plant-Derived Dietary Protein

The quality of a dietary protein is primarily characterized according to its content of EAAs (24). It is often measured using the Digestible Indispensable Amino Acid Score (DIAAS), by which the quality of a protein is considered according to its digestible AA content, compared to a reference protein and its ileal digestibility (24, 25). When using the DIAAS score, the following classification of protein quality is used: <75 no quality claim; 75–99 corresponds to high-quality protein, while a DIAAS score >100 corresponds to excellent protein quality (24, 25). Although various plant food sources contain considerable amounts of dietary protein, plant-derived proteins are commonly considered of lower quality and lower nutritional value than proteins from animal-derived sources based on its DIAAS score, as shown in Table 1.

**Table 1:** Protein quality (measured by the “Digestible Indispensable Amino Acid Score”, DIAAS) of selected animal and plant protein sources.

**Sources**	**DIAAS Score**
**Animal sources**	
Whey	85
Casein	117
Egg	101
Pork	117
**Plant sources**	
Soy	91
Hemp	54
Pea	70
Oat	57
Fava beans	55

*Adapted from: Herreman et al. (24)*.

Table 1 shows how the protein quality of common plant protein sources is considered less optimal compared to animal-derived protein sources based on its DIAAS score. This may also explain why people who only consume plants or plant-derived foods are generally known to have a lower intake of AA, including lower plasma concentrations of EAA than vegetarians, fish- and meat-eaters (26). Due to the lower protein quality of plant proteins, their ability to prevent whole-body protein deficiency and stimulate muscle protein synthesis (MPS) has been suggested to be lower compared to several animal-derived proteins (27). MPS stimulation may be crucial to facilitate the preservation of lean body mass and physical functioning, which is highly relevant in patients at increased risk of malnutrition, such as IBD patients (18, 27). In this context, studies assessing the postprandial MPS response of, for instance, soy protein have shown that ingestion of this protein does not increase MPS to the same extent as isonitrogenous amounts of common animal-derived protein sources such as whey protein, beef, or skimmed milk (27). In addition, beyond the EAA content of a protein source, the protein quality and the ability to stimulate MPS also depend upon the dietary protein digestion, including the AA absorption kinetics of the food sources (27, 28). Although it recently has been argued that there is limited evidence from human trials to support a considerable difference in protein digestibility between plant and animal protein sources, protein-derived from plant food sources may, in general, have lower digestibility compared to animal proteins (29). This depends, however, on the presence of factors that may influence the digestibility, e.g., preparation and cooking, the presence of components that interfere with the digestibility of the protein such as enzyme inhibitors, tannins, NDCs, and phytates, etc., which is found in some plant foods such as beans, cereals, and pulses (29–31). In other words, the proportion of AAs utilized and available for protein synthesis may be lower for plant-derived protein than protein for animal-derived foods (27). Moreover, it has been argued that protein-derived AAs from plant-based protein sources are converted to urea more rapidly when compared to animal proteins, which may decrease their ability to stimulate the anabolic response (27). Based on the above, there may be considerable drawbacks regarding ensuring sufficient intake of high-quality dietary protein when eating a plant-based diet compared to eating a diet that includes considerable amounts of animal-derived foods. However, it has been argued that the insufficient content of EAA in plant-based protein may be counteracted through consumption of a larger amount and variety of plant proteins per meal, which consequently leads to greater ingestion of EAA to cover dietary requirements (29). Similarly, increasing the amount of protein intake through increased consumption of plant-based protein sources may also compensate for the lower anabolic properties of plant-derived protein compared to protein from animal-derived foods (27). However, although increased consumption of plant-derived protein per meal may be an appropriate solution to minimize the difference between animal-derived protein and plant protein in relation to EAA consumption, the feasibility of such intervention may be limited, at least for some patients with IBD, due to IBD-related symptoms such as anorexia. To our knowledge, no studies have examined the feasibility and the impact on the nutritional status of such dietary intervention in IBD patients. Therefore, whether consumption of greater amounts of plant-based protein per meal is feasible must be determined based on an individual assessment of the patient according to whether the patient experiences IBD-related symptoms such as anorexia, including the risk of malnutrition.

### Microbial Fermentation of Dietary Protein

Although greater consumption of plant-based protein sources may fulfill the daily EAA requirement, excessive consumption of protein including its potentially limited digestibility may result in increased accessibility of peptides and AAs for microbial fermentation in the large intestine (32). In addition, insufficient absorption of protein in the small intestine may also occur in patients suffering from inflammation in the small intestine, as the ability to absorb nutrients may be compromised, which may also increase the risk of malnutrition (17, 18).

### Microbial-Produced Metabolites From Bacterial Protein Catabolism and Their Effects on Intestinal Homeostasis and Disease Activity in Patients With Inflammatory Bowel Disease

An increase in dietary protein consumption increases the amount of protein, peptides, and AAs that reach the colon to be metabolized by the colonic microbiota, resulting in microbial species changes (33, 34). While sulfur-containing AAs represent a powerful part of the cell antioxidant system, increased protein fermentation within the large intestine may have a detrimental impact on the gut barrier function, gut immune system, and intestinal inflammation through the production of several metabolites. These metabolites include hydrogen sulfide (H_2_S), aromatic compounds (e.g., phenols, P-cresol), polyamines including ammonia, and nitric oxide (NO), and studies have found a higher fecal concentration of several of these metabolites (e.g., ammonia and total sulfide) in IBD patients compared to healthy individuals (34). These protein-derived metabolites may contribute to intestinal inflammation through several mechanisms, as highlighted in recent reviews (32, 35). For example, H_2_S is produced through fermentation of several sulfur-containing AAs of dietary and endogenous proteins. In plants, the major sulfur AAs are cysteine and methionine, but the soluble fraction of the plants also contains a variety of other sulfur-containing compounds. Among other mechanisms, excessive luminal concentrations of H_2_S have been associated with increased expression of pro-inflammatory cytokines, including genes that may be involved in inflammatory processes of the intestinal epithelial cells (IECs) (32). These observations may explain why increased luminal concentrations of H_2_S have been associated with the etiology of UC (35). Furthermore, the production of aromatic compounds, e.g., indoles, branched-chain fatty acids, and biogenic amines, through microbial fermentation of aromatic AAs has also been suggested to impair the mucosal barrier through an increased epithelial permeability. Similarly, ammonia is suggested to produce damage to the mucus layer of the colon and interfere with the energy metabolism of the colonocytes. Moreover, this metabolite has also been shown to inhibit the oxidation of SCFAs such as in the colonocytes (32, 35). Although the metabolites primarily have been considered detrimental for intestinal health as described above, several of these substances have also been suggested to exert beneficial effects on the gastrointestinal system. For example, as highlighted by Yao et al. (32), H_2_S may be involved in anti-inflammatory processes in the colon. Similarly, fermentation of AAs in the large intestine gives rise to branched-chain fatty acids, i.e., isobutyrate and isovalerate, that may act as energy substrates for colonocytes when insufficient concentrations of butyrate are available as butyrate seems to be the preferred energy sources for the colonocytes (36). Despite the potential beneficial effects of some of these metabolites, the suggested detrimental effects of protein fermentation may partly explain why Western diets high in meats have been associated with a higher risk of IBD (37). In a prospective cohort study, a high intake of animal-derived protein, including meat and fish, was associated with an increased risk of IBD, however, no association was found between vegetable protein and IBD (38). Similarly, in a recent study, animal protein but not vegetable-derived protein was associated with risk of IBD. Moreover, meat and red meat consumption were found to associate with IBD risk. However, no association was found between total protein intake and IBD risk. In separate analyses on UC and CD, total meat and red meat were associated with risk of UC, while no associations were found for risk of CD (39). The suggested association between animal protein and IBD may be due to the higher content of sulfur and aromatic containing AAs in some animal proteins compared to plant proteins, which are metabolized into sulfur and phenolic compounds (32). In fact, a high intake of dietary sulfur has been associated with an increased risk of relapse of UC (40). Moreover, reducing dietary sulfur AAs from animal and plant foods may lead to clinical improvements in UC patients (34). However, the dietary guidance from the International Organization For The Study Of Inflammatory Bowel Disease (IOIBD) does not recommend reducing moderate consumption of unprocessed red meat including lean chicken meat and eggs in CD patients. In contrast, the IOIBD recommends reducing intakes of red and processed meat in UC patients. However, the evidence behind this recommendation was considered low (41). Hence, it remains to be determined if intakes of animal-derived protein are associated with risk of IBD and disease activity in IBD patients.

### Factors Influencing the Degree of Microbial Protein Fermentation in the Colon

Although there may be several detrimental effects of over-eating protein on intestinal homeostasis, caution must be drawn before neglecting proteins derived from plant foods despite increased requirements to ensure sufficient EAA. Several factors influence the rate of protein fermentation, such as the substrate availability (e.g., the AA composition of the digested protein sources), pH, and the ratio between carbohydrates and proteins within the diet. As described by Yao et al. (32), consumption of NDCs such as non-starch polysaccharides, resistant oligosaccharides, and resistant starch (RS) modulates the degree of protein fermentation, including the production of AA-derived metabolites. The suppressing effect on protein fermentation by NDCs has been suggested to result from a decreased demand of AAs used as energy sources by the microbes to support bacterial growth and lower pH due to a higher concentration of SCFAs within the gut (42). Together these factors cause an increase in saccharolytic fermentation and a decreased proteolytic fermentation (43). Moreover, different NDCs seem to have differential effects on protein fermentation depending on the fermentability of the NDC (32). As described in the section on carbohydrates (see below), different NDCs may have distinct physiological properties depending on their physiochemical structure. NDCs such as inulin and RS are fermented in the proximal colon compared to other NDCs (32). Therefore, the presence of these carbohydrates in the distal area of the colon is limited, resulting in a high protein fermentation (32). On the other hand, the inclusion of NDCs such as cellulose, which is only partially fermentable by the microbiota, is fermented in the distal colon where the bacterial density is higher compared to the proximal colon. This may result in a decreased fermentation of proteins due to the higher content of carbohydrates in this part of the gut (32, 44). For this reason, the inclusion of a variety of NDCs with varying physicochemical properties in the diet may be important to downregulate the rate of microbial fermentation of dietary proteins.

### Summary

To sum up, IBD patients may have an increased protein requirement depending on their disease status (active vs. remission). Adequate amounts of plant-derived protein may provide adequate amounts of AA and EAA essential for MPS and preservation of lean mass, which is particularly relevant to IBD patients as these patients are often at increased risk of malnutrition. However, high intakes of dietary protein may contribute to alterations of the gut microbiota and colonic inflammation by producing harmful metabolites. These effects may be attributed to animal-derived protein due to its high content of sulfur—and aromatic-containing AAs. Further, the potentially detrimental effects of increased protein consumption may, at least in part, be attenuated due to the coexisting consumption of various NDCs often found in plant-protein sources. However, increased consumption of plant-based foods to ensure proper intake of EAAs may not be feasible in all IBD patients due to IBD-related symptoms such as anorexia. In fact, at present, little is known about the role of proteins originating from plants in regard to actual measures of disease activity and nutritional status of IBD patients, as most of the research seemingly has been conducted in other populations or using animal protein. Thus, the role of plant-derived protein on IBD-related outcomes (i.e., measure of disease activity and nutritional status) remains to be determined.

## Plant-Derived Fatty Acids

Two factors determine the role of lipid nutrition in health and disease: (1) the composition and (2) the total amount of fat in the diet (45). Fatty acids play significant roles in relation to mucosal immune responses, the epithelial barrier function, oxidative stress, and inflammatory reactions and have an overall major influence on gut function and health (14). The composition of fatty acids and their molecular structures (chain length and number and position of double bonds) influence digestion, absorption, and metabolism of dietary fats and the bioactivity of the fatty acids. The fatty acid composition of the tissues and intestinal immune cells is highly influenced by the dietary fatty acid composition. Dietary fatty acids can therefore be a tool to manipulate inflammation reactions.

### Characteristics of Plant-Derived Dietary Fatty Acids

The quantitatively most important lipid component in the human diet is the triglyceride (TG) fraction, which accounts for more than 95% of the dietary fat. The dietary fatty acid composition of this fraction is highly dependent on the origin of the food, i.e., in general, the fatty acid composition of animal-derived food items consists mostly of SFAs, while plant-derived foods are rich in unsaturated fatty acids. With regard to IBD, it is generally said that people who eat more fruits and vegetables have a lower risk of IBD, while people who eat fewer fruits and vegetables and more animal fats and sugar have a higher risk of IBD (46). The suggested association between fat consumption, particularly trans-unsaturated fatty acids, n-6 fatty acids, and risk of IBD has been most evident for UC although the results of the existing studies are conflicting (46). However, one should be aware that the fatty acid composition of animal-derived foods varies a lot depending on the origin and type of product. According to Jakobsen et al. (47) the fatty acid profile of lean meat from the most commonly used food-producing animals amounts to 33–46% SFAs, 26–47% monounsaturated fatty acids (MUFAs), and 10–38% polyunsaturated fatty acids (PUFAs) of the total fatty acids (47). Milk fat contains about 60% of the total fatty acids as SFA, 22% are MUFAs, and 3% are PUFAs. Because of the hydrogenation processes in the ruminants, the milk fat also contains trans fatty acids and conjugated linoleic acid (CLA), amounting to 5.8% and 0.8%, respectively (47). The hens' feed highly influences the fatty acid composition of the fat in egg yolk. Notably, the level of unsaturated fatty acids can be raised considerably. Many studies have been conducted to enrich eggs with n-3 fatty acids using fish oil, specific sources rich in EPA or DHA, or flaxseed (48). Likewise, the fatty acid composition of the body fat and tissues (including the gut) of monogastric animals and humans is influenced by the dietary fatty acid composition (14). Hence, animal-derived fat in foods is not always rich in SFAs as the fatty acid composition is highly influenced by the animal species, the rearing system, and the dietary fatty acid composition of the feed (49). The triglyceride fat originating from plant foods is highly concentrated in vegetable oils, but also avocadoes, olives, and nuts are food sources rich in lipid and consist of especially oleic acid (C18:1) and the essential fatty acids (50). The essential fatty acids, linoleic (C18:2n-6) and α-linolenic acid (C18:3n-3), cannot be synthesized in the mammalian organism and must therefore be provided by the diet. These fatty acids are precursors for the longer-chain, higher PUFAs of the n-6 or ω-6 and n-3 or ω-3 fatty acid families formed in the tissues by chain elongation and desaturation processes. There are no interconversions between the n-6 and n-3 fatty acid families, but the presence of one of them may suppress the conversion of the other (51). Vegetable oils differ with respect to their fatty acid composition, especially the proportion of C18:2n-6 and C18:3n-3. As can be deduced from Jakobsen et al. (47), soya bean oil and corn oil (having a linoleic acid content of 500–600 g per kg dry matter), and palm oil (83 g C18:2n-6 per kg dry matter), contains a ratio of n-6 to n-3 fatty acids of 9.6, 56.9, and 12.4. It should also be noted that wheat and oat, which are common sources of dietary fiber, have ratios of n-6 to n-3 fatty acids of 23–26. Vegetable oils such as linseed oil, rapeseed oil, and hemp oil have a much higher proportion of C18:3n-3 giving rise to n-6 to n-3 fatty acid ratios of 0.3 to 3.2. Rye is also characterized by having a much lower n-6 to n-3 fatty acid ratio (= 8.3) than other grains. Some plant oils (Echium, hemp) have a high content of stearidonic acid (SDA, C18:4), an intermediate fatty acid metabolized from γ-linolenic acid. The content of SFA in vegetable oils is in the range of 9–15% and consists primarily of C14:0, C16:0, and C18:0. However, certain oils (e.g., oil from coconut or Cuphea seeds) are well-known for their content of medium-chained fatty acids (C10:0 and C12:0), which thereby give rise to a high proportion of SFA. Thus, dietary fat from plant-based foods may provide a wide range of fatty acids. When considering the dietary fatty acid composition in relation to human health and immunity, probably the most researched long-chain fatty acid (LCFA) group is n-6 and n-3 fatty acids. The ratio of n-6 to n-3 fatty acids in the diet appears to modulate inflammatory responses. According to Simopoulos 2008 (52), Western diets that typically have a ratio of 20:1 of n-6 to n-3 fatty acids have been shown to increase the production of pro-inflammatory mediators. In contrast, diets with a ratio of approximately 1:1 of n-6 to n-3 fatty acids are considered protective against inflammation (45, 52). Not only is the Western diet high in total fat, but it is also often high in n-6 fatty acids, i.e., the American diet contains 11–25 times more n-6 than n-3 fatty acids (53). Changing from an animal-based diet or a Western diet to a plant-based diet would automatically reduce the intake of SFAs and trans-fatty acids and could easily reduce the ratio of n-6 to n-3 fatty acids. However, what is characteristic for plant fat is the lack of the longer-chained polyunsaturated omega-3 fatty acids (DHA and EPA), which are found in high concentrations in marine food sources. The challenge regarding a plant-based diet is that the conversion of C18:2n-6 and C18:3n-3 to longer chain PUFAs is limited in humans, and DHA and EPA must therefore be provided through the diet to be incorporated into tissues and cellular membranes of the body. However, fish oil supplementation is an efficient way of providing EPA and DHA, and when provided to lung cancer patients, we have observed that the proportion of EPA and DHA in plasma phospholipids increased primarily at the expense of linoleic and arachidonic acid (14). Hence, consumption of plant-based diets will probably limit SFA consumption, and depending on the choice of plant foods, it may also be possible to obtain a low ratio of n-6 to n-3 fatty acids. Lowering the proportion of n-6 fatty acids in cellular membranes will reduce the pro-inflammatory eicosanoid synthesis of the n-6 series, which has been found to exert clinical efficacy in human inflammatory diseases (54). The so-called anti-inflammatory eicosanoids formed via the metabolism of EPA and DHA have very similar molecular structures as those originating from arachidonic acid in the n-6 series but with very different biological activity. However, diets of only plant foods lack EPA and DHA, and humans can only to a minimal extent metabolize these fatty acids from C18:3n-3. Maternal provision of SDA in form of hemp oil increases the plasma concentration of EPA and DHA in the offspring, which means that plant-derived foods rich in SDA may enhance the concentration of these anti-inflammatory fatty acids in plasma (55).

### Impact of Dietary Fatty Acids on Disease Activity in Patients With Inflammatory Bowel Disease

A major focus in relation to the impact of dietary fats on intestinal inflammatory diseases has been devoted to the difference between high and low dietary fat consumption as recently reviewed (56). However, as also mentioned in the study, the nutritional composition, particularly the fatty acid composition, has not been reported, except the role of SFAs. High intakes of SFAs have been suggested to increase the risk of flares in patients with UC in remission during treatment with aminosalicylates (57). However, long-term intake of higher levels of omega-3 fatty acids has been associated with a lower risk of UC (53). Most investigated is probably the impact of fish oil on disease activity in IBD, as EPA and DHA are well-documented to exert anti-inflammatory effects. Several systematic reviews have investigated the impact of n-3 fatty acids from fish oil on relapse and remission in IBD patients. De Ley et al. (58) evaluated the efficacy of n-3 fatty acids to induce remission in UC using all available randomized controlled trials. They concluded that there was no definitive conclusion regarding the efficacy of fish oil for induction of remission in UC patients and that adequate information is lacking to make recommendations for clinical practice (58). Similarly, in a Cochrane review, no evidence was found that supports the use of dietary supplementations of n-3 fatty acids for the maintenance of remission in UC patients (59). However, despite these results, the dietary guidance from the IOIBD (41) recommends increased consumption of natural sources of n-3 fatty acids (e.g., from wild salmon and other natural sources, not from supplements) for UC patients. The reason for pointing out that the fish should be wild or from natural sources is probably ascribed to the fact that wild salmon contains a much higher proportion of EPA and DHA compared to farmed fish as farmed fish is also fed vegetable oil rich feed, while wild fish eat krill and other marine EPA and DHA sources. With regard to Crohn's disease, a meta-analysis of six heterogeneous trials with 1,039 patients showed a small benefit of fish oil supplementation for reduction of relapse (60). However, in contrast, in two large randomized clinical trials, supplemental n-3 PUFA (from fish oil) did not prevent relapse of CD (61). Furthermore, in a study on enteral diets in CD patients, it was observed that patients receiving C18:2n-6 had higher remission rates than patients receiving oleic acid (62). A recent meta-analysis by Ajabnoor et al. (63) studying the long-term effects (more than 24 weeks) of increasing omega-3, omega-6, and total PUFA on IBD outcomes (including induced relapse or remission) and various markers of inflammation concluded that high intakes of long-chained n-3 fatty acids might reduce risk of IBD relapse, IBD worsening, and reduce the erythrocyte sedimentation rate, which together with C-reactive protein is a biomarker for systemic inflammation and a non-specific indicator of IBD. However, based on low-quality evidence, it was also suggested that long-chained n-3 fatty acids might increase the risk of IBD. Similarity increased fecal calprotectin was reported, which is a specific marker of IBD (63). Little or no effect was observed for C18:3n-3, omega-6, and total PUFA on IBD risk and relapses; however, data were sparse (63). Hence, it remains to be elucidated if the plant-based n-6 and n-3 fatty acids could influence the treatment of IBD and intestinal inflammation. When investigating the effect of dietary fatty acids on IBD and inflammation, the incorporation of fatty acids into the tissues, including gastrointestinal tissues of the human subjects, has not been analyzed, and several studies are performed without information on the bioavailability of the fatty acids. The omega-3 index is defined as the proportion of the sum of EPA and DHA content in the total fatty acids content in the erythrocyte membrane, and the omega-3 index is a good indicator of the incorporation of these fatty acids in gastrointestinal tissues (64). Several rodent and human studies have shown that diets rich in n-3 PUFA can reduce inflammation in the ileum and colon, in part, by reducing oxidative stress, modifying the gut microbiota and inflammatory reactions (56). Similarly, it has recently been shown that individuals having the highest relative arachidonic acid concentrations in adipose tissue had a significantly greater risk of developing UC (65). Modifying tissue fatty acid composition through diet might therefore prevent UC or reduce disease symptoms. Replacement of animal-based fat by adopting a plant-based diet with a high proportion of C18:3n-3 would result in an enhanced proportion of C18:3 in the gastrointestinal tissues on expense of C18:2n-6 and C20:4n-6. An increase in EPA level and a certain degree of DHA can probably be achieved by reducing the supply of linoleic acid and/or increasing the intake of α-linolenic acid (49). However, it should be stressed that C18:3n-3 is converted via enzymes (desaturases and elongases) to a minimal extent to EPA and DHA, i.e., 10-14% for men and women, respectively. However, there are large differences between individuals in obtained tissue C18:3n-3 levels after dietary supplementation, which may be ascribed to the metabolic interactions of individual omega-3 fatty acids (64). A study with colitis-induced rats found that C18:3n-3 decreased neutrophil function and infiltration and had a beneficial effect on colonic Inducible Nitric Oxide Synthase (iNOS) expression, glutathione (GSH) concentration and inflammatory stress (measured as reduced secretion of tumor necrosis factor (TNF) and mRNA level, cyclooxygenase (COX)-2 expression, lowered leukotriene B4 (LTB4) and Interleukin 6 (IL-6) production). In contrast, no effect on the peroxisome proliferator-activated receptor y (PPAR-γ) activation was observed (66). Some studies have observed exacerbating effects of flaxseed oil (being rich in C18:3n-3) on colitis in *C. rodentium* challenged mice despite increased n-3 PUFAs in intestinal tissues and increased caecal concentration of SCFAs (with proposed anti-inflammatory effects) (67). Other studies on plant oils have revealed that when mother rats during gestation and lactation were fed a diet high in safflower oil (around 72% C18:2n-6) compared to those fed diets high in canola oil (C18:3n-3) or high in oleic safflower oil (C18:1n-9), off-springs had more severe colitis (68). When using data from 7-day food diaries, it was also concluded that dietary oleic acid was inversely associated (while C20:4n-6 was positively correlated) with UC development (69). Dietary fatty acids, and the characteristics of the vegetable oils (e.g., the level of refinement), influence the intestinal microbiota, e.g., an altered n-6 to n-3 ratio have been shown to promote the presence of, e.g., *Enterobacteria* and *Clostridia spp*. leading to a pro-inflammatory environment that could be attenuated by including n-3 PUFA in the diet (70).

### Summary

To sum up, it remains to be elucidated if dietary plant-derived fatty acids would be beneficial in relation to disease activity in UC and CD patients. It seems most likely that reducing the SFA content and the level of n-6 fatty acids (relatively to n-3 fatty acids) in the diet by consumption of plant-based foods potentially could minimize disease activity via the incorporation of n-3 acids into the host membrane phospholipids or due to their influence on the intestinal microbiota. However, plant sources rich in n-3 fatty acids do not contain EPA and DHA, and although controversy exists regarding the influence of these longer-chained n-3 fatty acids on disease activity, a dietary change from an animal-based diet toward a diet primarily based on plant foods should not include omission of fish or other marine foods as the metabolism of n-3 fatty acids into anti-inflammatory longer chained fatty acids from plant sources are limited.

## Plant-Derived Dietary Carbohydrates

Plant-based foods represent a major source of dietary carbohydrates in the human diet, with a wide range of physiological properties important for human health (71). Many carbohydrates consumed from the diet are nutritionally defined as glycemic carbohydrates (GC) and include monosaccharides, disaccharides (i.e., sugars), malto-oligosaccharides, and polysaccharides (e.g., starch) (71, 72). GC is found in various foods including several plant-based foods as depicted in Table 2. GC is hydrolyzed into monosaccharides in the small intestine providing glucose for metabolism through peripheral circulation. In addition to GC, NDCs also represent a large group of carbohydrates in the human diet, and much interest has been devoted to the role of these carbohydrates in relation to the intestinal microbiota. NDCs consist of carbohydrates that, in general, are resistant to digestion and absorption in the small intestine (71, 72). As these carbohydrates resist digestion in the small intestine, they are metabolized in different parts of the colon and act as essential substrates for bacterial anaerobic fermentation, resulting in several microbiome-modulated postbiotics such as SCFAs (i.e., butyrate, acetate, and propionate) (11, 44, 76). NDCs are often referred to as “dietary fibers” and the majority of NDCs in the diet come from plant food such as fruits and vegetables as shown in Table 2 (71). The main types of NDCs include non-starch polysaccharides such as cellulose, hemicelluloses, pectins, and hydrocolloids. Other main types of NDCs are RS, resistant oligosaccharides such as fructooligosaccharides (FOS) and galactooligosaccharides (GOS), inulin, and other related substances such as lignin (71).

**Table 2:** Food sources of glycaemic- and non-digestible carbohydrates.

**Common types of carbohydrates**	**Examples of food sources**
**Glycemic carbohydrates**	
Starch	Cereals, potatoes, and bread
Sugars	Fruits and berries
**Non-digestible carbohydrates**	
**Non-starch polysaccharides**	
Cellulose, hemicelluloses, pectins, hydrocolloids: e.g. beta-glucans	High fiber grains and cereals, various fruits, vegetables and oats
**Resistant oligosaccharides and Inulin**	
Fructo-oligosaccharides, Galacto-oligosaccharides	Legumes and pulses, nuts, seeds, onions, garlic, Jerusalem artichoke, wheat, bananas, and leeks
**Resistant starch**	
Resistant starch	Oats, legumes, and unripe fruits like firm bananas, cooked and cooled potatoes

*Information obtained from: (73), (74), (71), and (75)*.

Although NDCs are generally considered dietary fibers, NDCs represent a heterogeneous group of carbohydrates of varying chemical structures with varying composition and branching patterns (72, 77). This causes variable physicochemical properties, site of utilization within the digestive system, and fermentation patterns resulting in different physiologic effects within the intestinal system (44, 72). As concluded in a recent review, the physicochemical characteristics (such as solubility, viscosity, and fermentability) drive different functionalities of dietary fibers in the gastrointestinal tract, which support their therapeutic potential (78).

### Non-digestible Carbohydrates, Postbiotics, and Intestinal Function

Due to their chemical structure, NDCs have different physiochemical properties influencing their impact on the gastrointestinal function. Dietary cellulose and certain hemicelluloses are generally considered insoluble in water, enabling them to increase the fecal mass and colonic transit rate by stimulating the intestinal mucosa (74). However, on the other hand, these NDCs are only partially fermented by the intestinal microbiota (44, 76). In contrast to insoluble NDCs, most NDCs that are considered soluble in water, such as FOS, GOS, inulin, pectins, and beta-glucans, are highly accessible for fermentation by the intestinal microbiota (76, 79). Through microbial fermentation, these microbiota-accessible carbohydrates can potentially impact gastrointestinal health by changing the structure and diversity of the microbiota by promoting the growth of microorganisms associated with intestinal health and increase the production of intestinal postbiotics such as SCFAs (80). Similarly, although often considered insoluble in water, RS is highly fermentable by the intestinal microbiota and gives rise to compositional shifts in the intestinal microbiota and considerable SCFAs production (44, 76). SCFAs, mainly acetate, propionate, and particularly butyrate, are suggested to play important roles in maintaining intestinal homeostasis and the regulation of intestinal inflammation (81). The concentration of these fermentation products varies along the length of the gut, and the highest levels of SCFAs are found in the cecum and proximal colon while the levels of SCFAs decline toward the distal colon (11, 81). Butyrate is absorbed and utilized in the proximal colon by epithelial cells and acts as the preferred energy source for colonocytes, whereas other absorbed SCFAs reach peripheral circulation to a varying degree (11). These SCFAs contribute to decreased pH levels in the lumen within the gastrointestinal tract. A decreased pH level has been suggested to inhibit the growth of pathogenic bacteria strains. Similarly, the decreased luminal pH level contributes to the production of intestinal mucins by the epithelial cells, while butyrate has been suggested to enhance the production of mucin 2, secreted by goblet cells which are important components of the mucosal barrier (7, 79). The mucosal barrier plays an important role in maintaining intestinal homeostasis, as it prevents translocation and colonization of microorganisms across the epithelium (7). Low consumption of NDCs leading to reduced SCFAs production may lead to impairment of the mucus layer due to a change in the microbial metabolism toward utilizing host mucins, dietary and endogenous proteins resulting in a reduced fermentative activity of the intestinal microbiota. This may result in an impaired mucosal barrier function (11, 79). Similarly, consumption of NDCs and the following production of SCFAs may also play a pivotal role in reinforcing the mucosal barrier function through the release of antimicrobial proteins (AMPs) by the IECs into the intestinal lumen, which prevents microorganisms from contact with the epithelial surface (7, 13). Moreover, butyrate has also been suggested to enhance the intestinal barrier function by induction of genes that encode tight-junction proteins and restoring hypoxia-inducible factor-1 (HIF-1) expression (13). Together, insufficient consumption of dietary NDCs may therefore compromise the mucosal barrier function, partly due to an altered production of SCFAs. Similarly, these observations also illustrate how NDCs may enhance the intestinal barrier function and reduce the epithelial permeability that contributes to mucosal inflammation and thus sustain intestinal homeostasis. SCFAs are also suggested to exert a direct anti-inflammatory effect in the intestinal mucosa through histone deacetylases (HDAC) inhibition and activation of SCFA-specific G-Protein-Coupled Receptors (GPCRs) that are expressed on IECs and immune cells (81, 82). Certain NDCs, such as FOS and GOS, have also been suggested to interact directly with the gut immune cells independent of the production of SCFAs (7, 73). Similar mechanisms have been described for other NDCs as pectin, which has been suggested to block pro-inflammatory pathways in human dendritic cells (13). Due to those mechanisms described above, NDCs and SCFAs may play a pivotal role in promoting or maintaining clinical remission in IBD patients. However, in IBD patients, decreased SCFA production may occur. Based on fecal samples of patients with IBD, the SCFA levels within the gut of these patients are often decreased due to alterations of the microbiota resulting in reduced microbial diversity. The concentration of SCFAs within the gut has also been suggested to be inversely associated with disease activity in UC patients in remission (7, 13). The microbial alterations observed in IBD patients are in general associated with increased levels of bacteria that possess pathogenic and inflammatory properties with the ability to adhere to the intestinal epithelium but also a decrease in the number of SCFA and dominant butyrate-producing bacteria such as *F. prausnitzii* and *Clostridium* cluster IV, XIVa, XVIII (83). In CD patients, alterations in butyrate-producing bacteria have been demonstrated, such as *Roseburia inulinivorans, Ruminococcus torques*, and *Clostridium lavalence* compared to healthy individuals (83). Similarly, alterations of butyrate-producing bacteria in UC patients have been reported, such as *Roseburia intestinalis* and *Roseburia Hominis* (13). Moreover, the presence/absence of *F. prausnitzii* has been associated with disease status in IBD (13, 83). The increase in pathogenic bacteria that impact the intestinal epithelium is thought to induce inflammatory responses by regulating the expression of several genes involved in the induction of intestinal inflammation (83). Collectively, these observations indicate that the microbial alterations, which lead to impaired production of SCFAs observed in IBD patients, may profoundly impact the course of disease in these patients. As NDCs give rise to microbial growth and compositional shifts of the commensal bacteria within the gut, intakes of NDCs may have significant potential to influence the activity of the disease in IBD patients by enhancing the abundance of SCFAs through the provision of substrates for microbial fermentation and the enrichment of postbiotic producing bacteria. This stimulation of growth of microbes within the gut and the production of SCFAs result from cross-feeding mechanisms, substrate-specific shifts, and both broadly and selective fermentation patterns of NDCs (44, 76). For example, dietary consumption of RS has been found to cause proliferation of *Bifibacterium, R. bromii*, and *Eubacterium rectale* depending on the type of RS consumed (84). Similarly, the consumption of inulin-type fructans such as inulin and FOS, commonly referred to as prebiotics, are suggested to increase the abundance of *Bifidobacteria* and *lactobacilli* that mainly produce acetate and lactate (85). Moreover, consumption of inulin-type fructans have also been suggested to cause both a bifidogenic and a butyrogenic effect through cross-feeding interactions between *Bifidobacteria* and butyrate-producing bacteria (86). Furthermore, consumption of pectin, a NDC commonly found in fruits and vegetables, has been suggested to increase acetate production, but only a minor proportion of propionate and butyrate. Similar results have been observed for cellulose, although this NDC is only partially fermented by the intestinal microbiota (44, 87). Based on these results, several NDCs may possess the ability to interfere with the microbiota, although some of these carbohydrates are considered only partially accessible for fermentation (80). This observation has also recently been highlighted by others, who have stressed that nearly all NDCs can induce specific shifts in the composition of the intestinal microbiota, which may contribute to several health benefits (79). Together, these observations support the fact that the enrichment of bacterial taxa, the production of the total amount and proportion of individual SCFAs is, at least to some extent, NDCs dependent. Still, almost all NDCs may have the ability to interact with the microbiome, which supports the inclusion of various NDCs in the diet (44). Similarly, plant foods high in NDCs do often contain a complex mix of different NDCs. Thus, from a practical point of view, consuming a variety of plant foods high in NDCs may be preferable. In addition, the potential effects of plant foods on IBD may also relate to its anatomic site of utilization. As most NDCs undergo fermentation in the colon, the therapeutic effects of these carbohydrates, including the SCFAs produced by NDC fermentation, may be of more relevance for patients suffering from UC than CD patients (44). However, as short-chain carbohydrates such as FOS and GOS, but also pectins are metabolized in the ileum and ascending colon, these carbohydrates may, in particular, possess the physiological potential to attenuate intestinal inflammation in patients with CD in this area of the gut (44, 74).

### Non-digestible Carbohydrates and Their Influence on Disease Activity in Patients With Inflammatory Bowel Disease

Although there seems to be a mechanistic rationale for the role of NDCs in regulating intestinal inflammation through mechanisms related to the modulation of the intestinal microbiota and the production of SCFAs, human studies on NDCs in IBD are inconclusive. In a recent meta-analysis of observational studies on associations between NDCs consumption and risk of IBD, consumption of NDCs was associated with lower risk of IBD, and a linear dose-response relationship between intakes of NDCs and risk of CD was found (88). In contrast, in a recent prospective cohort study of 401,326 participants from eight European countries, no associations between consumption of NDCs and IBD were found (89). Similarly, in another recent meta-analysis of 14 case-control studies, higher intakes of vegetables were associated with a lower risk of UC but not CD. In contrast, higher consumption of fruits was inversely associated with UC and CD risk (90). Although both fruit and vegetables are generally high in NDCs, other nutrients found in these foods may explain these results and not the NDCs content *per se*. Nevertheless, these inconsistent results highlight that the role of NDCs and the risk of IBD is still a matter of debate and needs further investigations. In a systematic review of 23 randomized controlled trials investigating the impact of various NDCs on disease activity in patients diagnosed with UC, pouchitis, or CD in various state of disease (i.e., patients in remission, with active disease, or both), only limited weak evidence of the role of NDCs on disease activity was found (91). Of all the studies examining the effect of various NDCs on disease activity in CD patients, no study reported any positive effects on disease activity when the intervention group was compared to the comparator group (91). However, one study found that CD patients in remission who consumed a high-fiber diet had a shorter time to relapse than patients on a low fiber exclusion diet (91). In contrast, in a small open-label trial of patients with ileocolonic CD, supplementation with 15g/d FOS for 3 weeks resulted in reduced disease activity and increased numbers of faecal *Bifidobacteria* and mucosal dendritic cells (92). Similar results were found in a recent observational study in which CD patients in remission with NDCs intakes in the highest quartile had lower odds of experiencing flares compared to those patients with an intake in the lowest quartile. However, no association between NDCs and flare in UC patients was found in this study (93). Of the studies of UC patients included in the systematic review, 6 out of 10 studies reported on disease activity. In only three of these studies, dietary intakes of NDCs had positive effects on the activity of the disease (i.e., prolonged remission or prevention of relapse) (91). Similarly, in another study of UC patients, not included in the review, dietary intakes of germinated barley foodstuff (GBF) (20g/d for 12 months) which is high in NDCs prolonged the period of clinical remission in UC patients (94). Similar results were found in a multi-center trial that found that 20–30 g of GBF for 24 weeks significantly reduced the disease activity in UC patients with mildly to moderately disease activity at baseline (95). A comparable conclusion was also drawn based on the results of a double-blinded placebo-controlled trial of patients with an ileal pouch-anal anastomosis, also included in the review, in which dietary intakes of inulin for 3 weeks resulted in significantly lower histological and endoscopic disease activity, lower pH, and decreased numbers of *Bacteroides fragilis* including higher butyrate concentrations compared to placebo (91, 96). In the studies included in the systematic review, not reporting on disease activity, dietary administration of NDCs in UC patients resulted in lower gastrointestinal symptoms and beneficial effects on inflammatory markers (91). However, whether these effects are related to disease activity is unknown. Thus, based on these results, only suggestive evidence seems to exist for the efficacy of various NDCs on disease activity in IBD patients. However, considerable heterogeneity exists between the existing studies making it hard to compare their results. For example, different study designs have been used and patients with different disease stages were included across the studies. Similarly, different outcome measures, varying measures of disease activity, study durations, and amounts of NDCs have also been used across the studies (91–95). Furthermore, in some of the studies included in the review by Wedlake et al. (91), the NDC intervention was co-supplemented with probiotics (91). In addition, the few studies demonstrating significant effects on disease outcomes included in the review by Wedlake et al., (91) used NDC supplementation rather than dietary interventions (91). As highlighted by Wedlake et al. (91), these results could be due to several reasons. For example, intakes of NDC supplements compared to dietary interventions are rather different interventions. The NDC supplementation used in the studies included in the review consisted of NDCs with well-known fermentative properties that may give rise to microbial growth and considerable SCFAs production. In contrast, dietary interventions may be vulnerable to individual interpretations resulting in intakes of NDCs in varying amounts and with a varying degree of fermentability (91). In addition, most studies reporting a significant effect on disease outcomes were conducted in patients with intestinal inflammation located in the colon or at the ileocolonic area of the intestine. As highlighted by Wedlake et al. (91), these results, may therefore, at least in part, be attributed to the site of microbial fermentation, which is in the colon. Furthermore, many patients suffering from CD experience inflammation more proximally to the site of microbial fermentation. Therefore, this may have implications for the results depending on the NDCs that have been consumed (44, 91). As highlighted above, NDCs such as FOS are utilized in the ileum and ascending colon, which may explain why Lindsay et al. (92) have reported decreased disease activity in CD patients with inflammation in this part of the gut consuming these carbohydrates (92). However, this study was a small open-label trial, and other studies of CD patients have found other results, making it difficult to draw a strong conclusion based on this result (97). Interestingly, although the studies of CD patients included in the systematic review reported no effects of NDCs on disease activity compared to placebo, within-group differences and differences between groups were observed in regard to the microbiota composition, SCFAs production, and gut immunoregulation in several of the studies (91). For example, in a RCT study of patients with active CD included in the systematic review, patients were supplemented 15 g FOS/d for 4 weeks, which resulted in increased IL-10 and reduced IL-6 positive lamina propria dendritic cells compared to placebo. However, no differences were observed in disease activity (97). Based on these observations, microbial and immunomodulatory properties of consumption of NDCs may not necessarily associate with measures of clinical disease activity in IBD patients. Although the results of the existing studies on NDCs consumption and disease activity in IBD patients are inconclusive, the IOIBD recommends moderate to high consumption of fruits and vegetables (commonly high in NDCs) for CD patients without symptomatic or significant fibrostricturing disease. For UC patients, no specific change or restriction in fruit and vegetable intake is recommended (41). Therefore, the recommendation of foods high in NDCs must be evaluated based on a thorough nutritional assessment of the patient in which the presence of malnutrition, anorexia, the dietary intake of the patient, and the clinical disease activity is determined. However, in case of malnutrition and poor appetite, it may be reasonable to decrease consumption of foods high in NDCs and encourage patients to eat foods of low volume, but high in calories despite this may result in a reduced intake of NDCs.

### Summary

In summary, plant-based foods are a key source of dietary carbohydrates in the human diet and provide large amounts of NDCs that have been suggested to play a significant regulatory effect on the gut. However, although mechanistic links have been proposed linking consumption of NDCs to reduced disease activity in IBD patients, there seems to be only limited evidence available on the impact of NDCs on disease activity from clinical trials. Therefore, more research is needed to determine the role of these carbohydrates in managing disease activity in IBD patients.

## Plant-Derived Phytochemicals and Inflammatory Bowel Disease

Compared to animal-based diets, consumption of diets rich in plant-based foods contributes to a high intake of phytochemicals, and one serving of a plant-based meal has been suggested to provide approximately 25,000 different phytochemicals in small amounts of each (75). Phytochemicals are a large group of non-nutrient metabolites that occur naturally in all plants and plant foods. Phytochemicals are synthesized by plants as secondary metabolites and possess important biological functions in the plant. These chemicals protect against herbivores and microorganisms while controlling important functions in relation to plant growth and reproduction (98). In addition to their biological functions in plants, phytochemicals may also have beneficial health effects with potential therapeutic implications for IBD through multiple mechanisms. These compounds may influence intestinal health through their ability to interact with the composition and function of the intestinal microbiota. Studies have demonstrated a mutual relationship between the intestinal microbiota and several phytochemicals. The gut microbiota metabolizes phytochemicals while these substances, including its derived metabolites, may modulate the composition and function of the microbiota (99). Similarly, these bioactive components and their metabolites are believed to have immune regulatory effects, including strong anti-oxidative and anti-inflammatory properties through their regulatory functions on various enzyme and cell receptors (100–102).

### Polyphenols

Among phytochemicals, particular interest has been devoted to plant-derived polyphenolic compounds found in abundance in the human diet (103, 104), and more than 8,000 chemical compounds from the plant kingdom have been isolated and described (105). Polyphenols define a large group of heterogeneous compounds classified into flavonoids or non-flavonoids depending on their chemical complexity and structure. Both flavonoids and non-flavonoids can be further subdivided into several classes and subclasses of these compounds, as shown in Table 3.

**Table 3:** Classification of polyphenols.

**Class name**	**Examples of derivatives**
**Flavonoids**	
Flavonols	Kaempferol, Quercetin, Myricetin
Flavones	Apigenin, Luteolin
Isoflavones	Daidzein, Genistein
Flavanones	Naringenin, Eriodictyol, Hesperetin
Anthocyanidins	Pelargonidin, Cyanidin, Delphinidin, Petunidin, Malvidin
Flavanols	Catechins, Gallocetechin
**Non-flavonoids**	
Hydrobenzoic acids	Protocatechuic acid, Gallic acid
Hydroxycinnamic acids	Coumaric acid, Caffeic acid, Ferulic acid, Curcumin
Stilbenes	Resvertrol
Lignans	Secoisolariciresinol

*Adapted from Hardman et al. (106)*.

### Polyphenol Content in Plant Foods

Polyphenols are widely present in various parts of vegetables, spices, and fruits, including roots, leaves, and seeds (107). Polyphenols also occur in other plant-derived food products such as tea and wine (107). From Table 4, the estimated polyphenol content of the 10 richest plant-based dietary sources of polyphenols is shown. As depicted, spices and herbs represent the richest sources of polyphenols. Other important sources of polyphenols include dark-colored berries, cocoa, chocolate, some seeds, and nuts. Despite not being listed in Table 4, vegetables such as olives, onions, spinach, broccoli, carrots, and potatoes also contribute with large amounts of polyphenols (107). At present, no dietary recommendation for phytochemicals or polyphenols exists. However, a recent study found that the highest daily mean intake of polyphenols in European populations was found in Denmark (men: 1,786 mg/day; women: 1,626 mg/day), whereas the lowest intake was found in Greece (men: 744 mg/day; women: 584 mg/d) (108). Thus, the highest daily mean intake of polyphenols found in Denmark corresponds to the content of polyphenols of approximately 100 g of black chokeberry. Although the foods shown in Table 4 are the primary sources of polyphenols in the diet, the concentration, properties, and composition of polyphenols in various foods differ considerably depending on the ripeness of the plant at the time of harvest, productions methods (e.g., conventional vs. organic production methods), environmental factors (e.g., soil type, exposure to sunlight, etc.), the food matrix (e.g., the presence of other micro-and micronutrients within the food) and food processing such as cooking (103). For example, peeling of fruits and vegetables may have considerable influence on the bio-accessibility (i.e., the amount of substrate available for absorption and utilization) of the polyphenol content of these foods, as the concentration of polyphenols often are higher in the outer part compared to the inner parts of the fruit. Thus, the concentration of phytochemicals consumed may be higher for non-peeled fruits compared to processed foods. Moreover, cooking and boiling have been shown to reduce the polyphenol content of foods (103). The polyphenol content of organically produced plant foods has also been demonstrated to be higher compared to conventionally grown crops (109). This has recently been shown in a systematic review and meta-analysis, in which substantially higher concentrations of several phenolic compounds were found in organic crops and crop-based foods (110). Together, these examples stress that several factors significantly influence the degree of bio-accessibility of polyphenols. These factors may ultimately affect the amount consumed depending on food origin, environmental circumstances during growth, and the used food preparation methods.

**Table 4:** Plant-based foods with a high content of polyphenols.

**Plant food sources**	**Content (mg/100 g or mg/100 ml)**
Cloves	15,118
Peppermint	11,960
Star anise	5,460
Cocoa powder	3,448
Mexican Oregano, dried	2,319
Celery seed	2,094
Black chokeberry	1,756
Dark Chocolate	1,664
Flaxseed meal	1,528
Black elderberry	1,359
Chestnut	1,215

*Adapted from Pérez-Jiménes et al. (107)*.

### Bio-accessibility and Bioavailability of Polyphenols: Absorption in the Small Intestine, Interaction With the Gut Microbiota and Intestinal Immunity

The impact of polyphenols on intestinal health may be closely related to both the bio-accessibility and bioavailability of the polyphenols, which generally is considered low (i.e., limited absorption and availability of polyphenols for metabolism) (103). The intestinal microbiota seems to play a significant role in the modulation of the bioavailability and production of polyphenol-derived metabolites as only small amounts of dietary polyphenols, and mainly those of less complex chemical structure (~5–10%), are absorbed in the small intestine (99). After absorption within the small intestine, these substances undergo Phase I metabolism (oxidation, reduction and hydrolysis) and Phase II metabolism (conjugation) in the enterocytes and the liver, which results in several polyphenol-derived metabolites that enter systemic circulation (99). The majority of polyphenols [90–95% of the total polyphenol intake (99)] are, however, not absorbed in the small intestine and these unabsorbed complexes reach the colon in which the intestinal microbiota plays an important role in the biotransformation of these compounds into several less complex phenolic compounds and polyphenolic-derived metabolites that often have higher bioavailability and bioactivity (111). For example, a common anthocyanin, i.e., cyanidine-3-glycoside, which during catabolism in the intestine form several bioactive phenolic compounds (such as ferulic acid, vanillic acid, protocatechuic acid and phloroglucinaldehyde) enhances the bioavailability of cyanidin-3-glucoside (112). These metabolites are available for further absorption or excretion via feaces. However, during their presence in the gut, the metabolites may also interact with the microbiota and/or host immune cells. Upon metabolism by the microbiota, the polyphenolic-derived metabolites undergo phase II metabolism in the colonocytes and the liver before entering systematic circulation and distribution to organs and tissue or urinary excretion (99). Through their metabolism within the intestinal microbiota, polyphenols and the polyphenols-derived metabolites alter the composition and diversity of the microbiota. Moreover, these polyphenolic compounds have bactericidal and bacteriostatic properties through their ability to bind to bacterial cell membranes and thus limit the adhesion of pathogenic bacteria to the intestinal epithelium, contributing to reduced intestinal inflammation (83, 99, 102). These compounds may alter the intestinal microbiota composition by increasing the microbial diversity and inhibiting the abundance of pathogen-associated bacteria while enhancing the proliferation of beneficial bacteria such as *Bifidobacterium, Lactobacillus*, and *F. prausnitzii* that are associated with anti-inflammatory properties through its butyrate-producing capacity (83, 111, 113). Intestinal dysbiosis, commonly characterized by a decreased abundance of bacteria belonging to *Clostridium* cluster IV and increased levels of mucolytic and pathogenic bacteria such as *Proteobacteria* and *Bacteroidetes*, is frequently observed in IBD patients and believed to play a pivotal role in its pathogenesis (83). Consumption of polyphenols may therefore have the ability to play a significant role in the restoration of intestinal dysbiosis by stimulating the growth of beneficial bacteria, which may result in hampered intestinal inflammation, which also was shown in a recent review on interactions between polyphenols and the intestinal microbiota in IBD (114). In addition to the potential impact on intestinal dysbiosis, polyphenols and polyphenolic-derived metabolites also exert important immunomodulatory effects. In a recent review summarizing the results of several *in vitro* cell lines studies and *in vivo* animal experimental models, Hossen et al. (115) described how phytochemicals and, in particular, polyphenols may alleviate IBD through mechanisms related to the inhibition of pro-inflammatory cytokines, oxidative stress amelioration and restoration of the intestinal mucosal barrier to prevent translocation of microbial products and antigens from the intestinal lumen to the lamina propria (7, 115). These bioactive substances are suggested to suppress the secretion of proinflammatory cytokines such as IL-6 and TNF which have been shown to be upregulated in IBD patients and increase the production of anti-inflammatory cytokines (115). Moreover, polyphenols have also been shown to reduce colonic myeloperoxidase (MPO) activity, a marker of oxidative stress, and mediate inflammatory cytokines. Oxidative stress resulting from excessive continuous production and release of Reactive Oxygen Species (ROS) by immune cells is coupled to chronic inflammation, and their release in actively inflamed mucosa is associated with intestinal dysbiosis and increased intestinal tissue breakdown (115, 116). The antioxidative properties of flavonoids have been subject to substantial attraction and several papers regarding flavonoids have been published in relation to nutrition of monogastrics (105). Furthermore, polyphenolic compounds may also reduce the activity of inflammatory mediators such as nuclear factor kappa-light-chain-enhancer of activated B-cells (NF-kB). NF-kB plays a critical role in signal pathways involved in inflammation, and its suppression has been demonstrated to result in a decreased production of pro-inflammatory cytokines in epithelial cells and macrophages (115). Despite these mechanisms, it is important to note that the doses of polyphenols used in these studies varied considerably and may not translate directly into human consumption dosage. Furthermore, the doses may not be possible to eat from regular foods and diets (117). Similarly, to our knowledge, the actual intake of polyphenols among IBD patients is largely unknown. Moreover, it may also be difficult to analyze the actual intake due to the number of different constituents (>8,000) belonging to the group of polyphenols. Therefore, it may be difficult to validate the existing results of the potential beneficial effects of polyphenols and their metabolites in clinical studies with the emphasis on determination of the effectiveness and safe dose of various polyphenols in IBD patients.

### Impact of Polyphenols on Disease Activity in Patients With Inflammatory Bowel Disease

While many studies have addressed the impact of polyphenols in relation to gut health of monogastrics, less studies have addressed their impact on human health and diseases [see for instance Cardona et al. (99)]. Most studies on various polyphenols in attenuating inflammation in IBD have been conducted in animal models, especially rodent models (117) and more recently the active plant phenolic acid protocatechuic acid was demonstrated to exert protective effects on oxidative stress, inflammation, and the intestinal barrier function in a pig model (118).

In clinical studies investigating the efficacy of polyphenols in IBD patients, promising results of polyphenols and phenolic compounds such as anthocyanin, resveratrol, epigallocatechin-3-gallate including foods rich in polyphenols (e.g., red wine, green tea and ginger) on various markers of inflammation, IBD-related symptoms and measures of disease activity have also been reported, although the number of studies is limited (119).

Despite the limited number of intervention studies investigating the efficacy of polyphenols in IBD patients, particular interest has been devoted to the hydrophobic polyphenolic compound curcumin, and to date, curcumin may be the most investigated polyphenol in IBD patients due to its potentially clinical therapeutic anti-inflammatory and immunoregulatory effects (119).

Therefore, the results of clinical studies investigating the efficacy of curcumin on measures of disease activity in IBD patients are summarized down below.

Curcumin has commonly been used in Ayurvedic and Chinese medicine. Curcumin is derived from *Curcuma Longa* and is the principal constituent of turmeric found in several common spices (120, 121). The impact of curcumin on intestinal inflammation has been the subject of several recent reviews. In summary, the effects of curcumin in IBD have primarily been attributed to its ability to reduce epithelial cell damage, suppress the expression of COX, prostaglandin E-2 (PGE2), including NF-kB activity that regulate proinflammatory cytokine gene expression (IL-1, IL-6, and TNF) in various cell types (122–125). Similarly, curcumin has been shown to suppress autophagy by down-regulating the expression of genes involved in the regulation of autophagy, which has been shown to attenuate inflammation (125). This is of particular relevance, as mutations in autophagy-associated genes are thought to result in translocation of pathobionts to the lamina propria, leading to intestinal inflammation in IBD (7). Although there seems to be a mechanistic rationale for curcumin in IBD treatment, results from clinical studies are few, and most studies have been conducted in UC patients.

In a recent systematic review of 6 placebo-controlled RCT studies of UC patients with a total of 372 participants with mild disease or disease in remission conducted by Coelho et al. (126), the efficacy of treatment with curcumin in combination with mesalamine (5-ASA) was investigated compared to placebo together with 5-ASA on disease activity (126). In most studies (*n*=4), oral capsule curcumin was used with a dosage ranging from 450 mg to 3 g/day (126). One of the included studies used a standardized extract with curcumin as an enema at a dosage of 140 mg/day, while another study used capsules of nano-micellar curcumin formulation with a dosage of 240 mg/day (126). Across the included studies, the duration of the interventions ranged from 4 weeks to 12 months (126). Clinical disease activity was measured using indices hereof and/or endoscopic measurements depending on the specific study. In five of the six included studies, significant clinical improvements measured by disease activity indices, including improved endoscopic activity, were observed with the curcumin intervention compared to placebo. Moreover, no side effects that could be related to curcumin were observed in the studies (126). Similar results were also found in a recent meta-analysis which also included the studies that were summarized by Coelho et al. (127).

In the sixth study included in the review by Coelho et al. (126), no significant difference between clinical and endoscopic remission rates of the intervention group and the placebo group was found. However, this study used significantly lower doses of oral curcumin (450 mg/day) than several of the other studies, which may indicate that the effect of curcumin is dose-dependent (126, 128). However, as also highlighted by the authors of the review, interestingly, in the studies, using either the enema with a dosage of 140 mg/day standardized extract with 72% curcumin or the nano-micellar curcumin formulation (240 mg/day), a significant difference in disease activity between the intervention and control group was found despite the use of lower doses compared to the other studies. This may stress the fact that the efficacy of curcumin may also depend on the administration route (e.g., locally administered through enemas) or the administration form (e.g., using more bioavailable products) (126). The latter may be relevant, as most polyphenols' bioavailability, including oral curcumin, is limited and high doses of curcumin are usually necessary to increase its bioavailability. Thus, higher doses of curcumin may be needed if administered orally compared to enema administration, where the curcumin is delivered directly to the distal gut (126). Moreover, only one of the studies included in the systematic review described the purity of the curcumin used, and therefore it is, in fact, unknown what the intervention groups were given in the remaining studies (126, 129). Similarly, it remains unknown whether the efficacy of curcumin depends on concomitant treatment with 5-ASA (126). Moreover, to our knowledge, no validated curcumin products are available at present, and it is questionable whether it is possible to consume sufficient doses of curcumin through the consumption of regular foods (e.g., spices) (126). Similarly, to our knowledge, it is unknown whether high intakes of foods to reach therapeutic amounts of curcumin may result in similar results as for curcumin itself or causes any side effects. Thus, despite promising results of curcumin, several questions still need to be answered. The number of studies is still limited, and the most optimal dose and administration route still need to be determined (126).

### Summary

In summary, the consumption of various plant foods provides many phytochemicals that may significantly impact intestinal health. Several fruits and vegetables contribute with considerable amounts of polyphenols, with spices and herbs as one of the richest sources. However, a substantial number of factors influence the actual intake of polyphenols, such as environmental factors (e.g., time of harvest, organic vs. conventional agriculture) and food processing methods such as cooking and boiling. Thus, the actual intake of polyphenols may vary considerably according to these factors. Moreover, the bioavailability of polyphenols may be limited. As only a limited number of studies have investigated the efficacy of polyphenols in IBD patients, the optimal safe dose and efficacy of these bioactive substances still need to be determined based on adequate powered clinical studies. Similarly, although clinical studies of curcumin in UC patients with promising results exist, the number of studies is limited. Likewise, the optimal dose and administration route still need to be determined. Furthermore, the efficacy of curcumin on disease activity in CD patients need to be elucidated. Thus, large RCT studies of curcumin in UC and CD patients are required to determine its efficacy.

## Vitamins and Trace Elements in Inflammatory Bowel Disease

Vitamins are organic micronutrients mainly synthesized by plants and do not provide energy. Vitamins are essential nutrients and humans are capable of synthesizing certain vitamins, while other vitamins cannot be synthesized by humans. Therefore, diets must supply the body with small amounts of vitamins daily to maintain the metabolic functions of the cells. It has often been stated that “healthy eating” would not require additional vitamin supplementation. However, the fact is that the majority of e.g., Americans do not follow a healthy eating pattern (130). Some vitamins are synthesized by human, e.g., vitamin D (in the skin after exposure to sunlight) and niacin (from tryptophan), and the colonic microbiota also synthesizes several of the B-vitamins, especially niacin and riboflavin, being the most commonly synthesized vitamins by the human gut bacteria (131, 132). However, it should be noted that it is not known how much the proportion of vitamins could be taken up by the non-producing microbiota. If the endogenous synthesis is not enough to cover daily needs, dietary intake is required, and due to the multiple impacts of vitamins on the gut function and health in monogastrics, vitamin nutrition requires special emphasis during inflammatory diseases (15). The requirement for vitamins by IBD patients may be higher than in the general population, and this patient group may be exposed to a higher risk of developing malnutrition, e.g., suffer from vitamin and mineral deficiency (18). It has been shown that IBD patients have a lower abundance of microbial enzymatic genes related to the biosynthesis of cobalamin and thiamine compared with healthy controls. This is consistent with results showing lowered levels of B-vitamins in plasma of IBD patients, which may be due to both decreased vitamin B absorption and microbial synthesis of B-vitamins in these patients (133). Furthermore, vitamin D status (measured as 25-hydroxyvitamin D in plasma) has been linked to risk of IBD, and IBD patients and especially CD patients appear to be at increased risk of vitamin D deficiency, especially those having small bowel resections (134). A recent review confirmed that vitamin D levels in serum were inversely related to CD and UC, and that these patients had lower serum levels of 25-hydroxyvitamin D than in healthy people, and that more than half of the patients had insufficient vitamin D levels (135). However, while deficiencies of vitamin D, B12, and other B-vitamins, are highly prevalent in patients with IBD, there is insufficient evidence to support the use of vitamins to induce or maintain remission in UC and CD (46). During the recent decade, especially vitamin D has been researched regarding its capability to induce and maintain alleviation of IBD through anti-bacterial and anti-inflammatory actions and repairment of the intestinal mucosal barrier (136). However, other micronutrients [vitamins and trace elements (selenium, zinc)] may also be highly relevant to consider in inflammatory diseases, given their multiple functions in inflammatory reactions, oxidative stress, and on the epithelial barrier function (15). Hence, future research should establish more fundamental knowledge of the requirement of micronutrients in IBD patients. In general, animal-derived food products provide good sources of various vitamins, including vitamins A and D, and B12, while vegetable fat contains vitamin E and carotenoids. Strict vegetarianism without adequate vitamin supplementation may lead to vitamin B12 deficiency because products of animal origin constitute the primary vitamin B12 source in the human diet. Since the vitamin D status is highly influenced by exposure to sunlight, food sources of vitamin D may become of special concern when the endogenous vitamin D synthesis is low due to a lack of sun exposure. Food items such as vitamin D enriched yeast and mushrooms could then be considered, and plant-based foods containing provitamin A, i.e., beta-carotene, while seafood (tuna, lobster, caviar) is sources of vitamin B12 (75). However, a transition from an animal-based to a plant-based diet to induce or maintain remission in IBD may call for a general vitamin supplement because it may be difficult or even impossible to cover the daily requirement of certain vitamins using plant-based foods only.

### Summary

To summarize, IBD patients may be at risk of developing deficiency for certain vitamins, i.e., vitamin D deficiency. Similarly, deficiencies of vitamin B12 and other B-vitamins are highly prevalent in IBD patients. While there is insufficient evidence to support the use of vitamins to induce or maintain remission in UC and CD, plant-based dietary habits may require vitamin supplementation of especially vitamin B12 and vitamin D.

## Conclusion and Future Research Directions

As discussed in this review, plant-based dietary components may impact various physiological mechanisms of intestinal inflammation. Therefore, the central question is whether it is possible to support and maintain clinical remission of IBD by adopting a plant-based eating pattern? Plant-based foods are major sources of NDCs and phytochemicals that are believed to interact with intestinal microbiota and exert direct influence on the gut immune system through several mechanisms related to the increased production of immunoregulatory postbiotics and downregulation of proinflammatory responses. That said, the clinical effects of NDCs and phytochemicals on induction and maintenance of disease activity in IBD patients remains inconclusive. Hence, more research is needed to determine whether these dietary components reduce disease activity in IBD patients. Although plant-based diets provide essential micronutrients, consuming a diet primarily based on plant foods may result in inadequate intakes of vitamin B12 and other micronutrients depending on the strictness of the diet, the nutritional status of the patient, and seasonal effects (regarding vitamin D status). Thus, vitamin supplementation may be needed. Consumption of plant-based diets, may also contribute to an increased dietary protein intake to ensure sufficient coverage of AAs and EAAs and the avoidance of malnutrition. Increased consumption of dietary protein may increase the amount of protein that reaches the colon, resulting in the production of several metabolites that may have detrimental effects on the gut and enhance intestinal inflammatory processes. However, whether plant and animal protein sources associate differently with IBD, and the course of disease, remains presently unknown. Furthermore, consuming foods primarily originating from plants may result in a decreased intake of SFAs and a more optimal ratio of n-6 fatty acids to n-3 fatty acids, which may decrease inflammatory responses and reduce disease activity in IBD patients. However, plant sources of n-3-fatty do not encompass EPA and DHA that are believed to exert anti-inflammatory properties. That said, most of the research on the role of EPA and DHA on disease activity has been conducted using fish oil with inconclusive results. Therefore, more research is needed to elucidate the role of these fatty acids and other fatty acids on disease activity in IBD patients. In the interim, adopting a diet primarily based on plant foods should not exclude fish and marine foods. To sum up, whether it is possible to induce and maintain clinical remission of IBD is not a simple “yes or no” question. Although the clinical effects of plant-based foods on disease activity need to be investigated further, clearly, plant-derived foods contribute with several food components and nutrients that may possess the ability to reduce and prevent intestinal inflammation. However, plant-based diets may also have drawbacks (e.g., potential difficulties in ensuring sufficient intakes of high-quality protein, longer-chained n-3 fatty acids, and some micronutrients) that need to be addressed. Moreover, IBD patients represent a diverse group of patients with distinct disease characteristics, of which the site of inflammation may be of utmost importance to consider with respect to the nutritional composition of the diet, and especially the specific role of bioactive components and their association with the locally present microbiome (see Figure 1). Besides, IBD patients are at increased risk of nutritional deficiency and malnutrition (e.g., vitamin-D and vitamin-B deficiency). Thus, it is our recommendation that a plant-based dietary intervention that aims to contribute to induction or maintenance of clinical remission in IBD patients should be designed based on an individual assessment of the patient in which the site of intestinal inflammation including the nutritional status of the patient, the presence of IBD-related symptoms, and other complications such as intestinal strictures are assessed.

## Author Contributions

CA, MH, HR, and CL: conceptualization and writing—review and editing. CA and CL: writing—original draft preparation. MH, HR, and CL: supervision. All authors have read and agreed to the published version of the manuscript. The authors contributed equally to the preparation of this manuscript.

## Funding

This research was supported by the Nutrition Board at Aalborg University Hospital, which provided funding for CA during his affiliation as a research assistant at the Department of Clinical Medicine, Faculty of Medicine, Aalborg University, Aalborg, Denmark.

## Conflict of Interest

The authors declare that the research was conducted in the absence of any commercial or financial relationships that could be construed as a potential conflict of interest.

## Publisher's Note

All claims expressed in this article are solely those of the authors and do not necessarily represent those of their affiliated organizations, or those of the publisher, the editors and the reviewers. Any product that may be evaluated in this article, or claim that may be made by its manufacturer, is not guaranteed or endorsed by the publisher.
